# A multimodal transformer-based visual question answering method integrating local and global information

**DOI:** 10.1371/journal.pone.0324757

**Published:** 2025-07-02

**Authors:** Cuiyang Huang, Zihan Hu

**Affiliations:** 1 Jinan University-University of Birmingham Joint Institute, Guangzhou, RP China; Tongji University, CHINA

## Abstract

Addressing the limitations in current visual question answering (VQA) models face limitations in multimodal feature fusion capabilities and often lack adequate consideration of local information, this study proposes a multimodal Transformer VQA network based on local and global information integration (LGMTNet). LGMTNet employs attention on local features within the context of global features, enabling it to capture both broad and detailed image information simultaneously, constructing a deep encoder-decoder module that directs image feature attention based on the question context, thereby enhancing visual-language feature fusion. A multimodal representation module is then designed to focus on essential question terms, reducing linguistic noise and extracting multimodal features. Finally, a feature aggregation module concatenates multimodal and question features to deepen question comprehension. Experimental results demonstrate that LGMTNet effectively focuses on local image features, integrates multimodal knowledge, and enhances feature fusion capabilities.

## 1. Introduction

The rapid development of multimodal visual-language tasks has attracted extensive attention within the Computer Vision (CV) and Natural Language Processing (NLP) communities, leading to notable advancements and emerging research areas in related task [1–4]. Visual question answering (VQA) poses huge challenge, as the model cannot identify the specific query content until it is input into the network, and different queries require diverse visual content; consequently, each object in an image varies in importance depending on the question.

Attention mechanisms have shown remarkable progress across deep neural network tasks, particularly within single-modality domains [5,6]. In VQA, utilizing attention on the question to capture main terms is crucial for improving answer prediction. Recent studies indicate that involving both visual and linguistic information within the network can facilitate a common representation, achieving finer-grained semantic expression and thus enhancing model performance and prediction accuracy [7]. However, these joint attention models have primarily captured only shallow interactions, with shared attention features lacking the complexity to support VQA model reasoning for intricate image interactions (e.g., object-question word relationships), thereby constraining model performance. To address this, researchers have proposed two joint attention models [8,9], which experimental results show are beneficial for understanding image-question relationships and enhancing VQA prediction accuracy. Notably, these models can be deeply cascaded, forming layered joint attention frameworks that support complex CV reasoning tasks, potentially boosting VQA effectiveness. Building on this, encoder-decoder structures based on attention have been designed to improve VQA model performance [10,11]. Despite advances in VQA models, existing approaches primarily focus on global feature interactions, resulting in erroneous predictions for queries that require only localized information.

Multimodal fusion of visual-language features represents the most direct approach to solving VQA queries. This involves first extracting global features of the image as well as from the question, then predicting the answer based on a multimodal fusion model that maximally integrates both types of information. Several methods have introduced increasingly sophisticated models for feature fusion, utilizing advanced question feature extractors, multimodal fusion models with residual connections, bilinear pooling, and guided attention mechanisms [8,12]. However, these approaches often apply question features to enhance visual attention, potentially over-emphasizing image features and overlooking the semantic essence of the question itself.

In summary, although recent methods have contributed to VQA model improvement, two main issues remain: (1) Current VQA models lack consideration for essential local image details during feature encoding, hindering accurate predictions for related queries; and (2) limitations in multimodal feature fusion impede fine-grained question comprehension, resulting in incorrect answer predictions.

To address these challenges, this study proposes a global-local attention (GLA) unit to emphasize prominent local visual features and integrates a self-attention (SA) module to model dense relations, such as word-to-word associations. A guided-attention (GA) unit is introduced to capture intensive cross-modal interactions between visual and linguistic features. Together, these three units form a foundational co-attention layer (CAL), with a deep vision-language encoding and decoding framework (D-VLEDF) constructed from stacked CAL layers. Additionally, multiple fusion strategies are applied during encoding and decoding to enhance question semantic comprehension, allowing the model to “see” relevant image features by using the query, and “hear” the query context in the multimodal representation module. The proposed network structure integrates these approaches to complete the VQA task effectively.

## 2. Related works

### 2.1 Methods based on multimodal feature fusion

VQA is a multimodal task requiring an understanding of both visual and linguistic knowledge. This involves mapping visual and linguistic features into a shared semantic space that captures the richness of multimodal knowledge. In early fusion methods, element-wise operations, such as multiplication, addition, or concatenation, are commonly used to combine visual and text features. Ren et al. [13] are the first to use concatenation to fuse visual and question features, aiming to learn a joint representation. They represented all question words through summation. Shih et al. [8] introduced an attention mechanism to select image regions relevant to the text query and map these visual and text features to a shared space, comparing their relevance through inner products. Similarly, Lu et al. [14] emphasized that focusing on both visual and textual attention is essential and proposed a joint attention model to reason over image and question attention to achieve a multimodal fusion representation. However, these methods have limited multimodal feature representation capabilities, constraining the model’s performance by restricting the amount of beneficial knowledge that can be derived.

To better model visual and linguistic features, researchers have proposed various fusion techniques. Kim et al. [15] and Li et al. [16] used element-wise multiplication for multimodal visual-language representation. Fukui et al. [17] suggested that the outer product of visual and text features is more expressive; however, its high dimensionality makes it impractical. Therefore, they proposed a two-stage compact bilinear pooling method that first computes attention for images and questions for joint representation, then integrates it with the question representation for efficient fusion. While effective, this approach is primarily suited for high-dimensional features. Kim et al. [18] addressed this limitation by using multimodal low-rank bilinear pooling with Hadamard products to reduce model parameters, although performance is sensitive to changes in hyperparameters. Building on this, Benyounes et al. [12] proposed a multimodal tensor Tucker decomposition, designing a low-rank matrix decomposition method to explicitly constrain the interaction rank between visual and text representations. Yu et al. [19] tackled the high-dimensional feature issue by integrating compact multimodal bilinear pooling and a joint attention mechanism [20], combines a “co-attention” mechanism with generalized high-order pooling for more discriminative image-question feature representation, improving multimodal fusion efficiency. Li et al. [21] employed contrastive loss with cross-modal attention alignment before fusion, enhancing visual-language feature fusion. Dou et al. [22] further improved VQA performance by inserting cross-modal attention within the visual and text encoding backbones.

### 2.2 Methods based on attention mechanism

Given their alignment with human cognition and interpretability, attention mechanisms quickly gained traction in VQA to advance research in this field. Yang et al. [23] proposed the stacked attention network (SAN), which incrementally searches for question-relevant image regions based on question semantics, enhancing accuracy by associating visual information with questions. Anderson et al. [24] used faster region-based convolutional neural network (R-CNN) [25] to extract multiple object regions from images, then applied a bottom-up, top-down attention mechanism to identify relevant image regions, capturing essential visual features. Schwartz et al. [26] introduced a general attention mechanism to learn high-order associations among data patterns. Recognizing that visual elements are often isolated in conventional attention mechanisms, Patro et al. [27] introduced attention weights based on different support and opposition samples, making attention more akin to human perception and aiding answer prediction. Nam et al. [28] proposed dual attention networks (DANs) to link specific image regions and question words through multiple steps, allowing visual and text attention to guide each other for collaborative reasoning, particularly effective in VQA. Peng et al. [29] proposed an attention framework that enhances connections between query keywords and image areas by simultaneously mapping relevant object regions and identifying associated words. These methods improve representation learning for both visual and textual modalities to better predict answers. Moreover, Nguyen et al. [30] proposed a simple yet symmetric architecture for visual and language representations, facilitating iterative interactions between image-question pairs. Gao et al. [31] introduced a dynamic approach to integrate intra- and inter-modal information flow, capturing higher-order interactions between questions and image regions to enhance VQA performance. Yu et al. [32] presented a multi-level, cascaded co-attention network to model intra- and inter-modal relationships. Guo et al. [33] calculated similarity between visual regions and question words, refocusing on image objects aligned with answers, reconstructing initial attention maps for consistent results. Tian et al. [34] developed a sequential top-down attention model to highlight essential details derived from both images and questions, suppressing irrelevant details, allowing more granular interactions for full multimodal fusion.

### 2.3 Reasoning-based visual question answering methods

Current VQA models typically focus on feature extraction without enhancing reasoning capabilities. In response, recent studies have developed reasoning mechanisms for processing extracted visual and linguistic features. Andreas et al. [35] proposed a neural modular network with modular layers (“Find, Transform, Combine, Describe, and Measure”) to predict answers, dynamically instantiating module networks based on question language substructures. Although effective, this method requires a natural language parse tree to generate substructures. Hu et al. [36] addressed this by proposing an end-to-end modular network that adjusts network structure and learns parameters without requiring pre-defined language substructures. Chen et al. [37] introduced a meta-modular network that dynamically configures function modules for complex visual reasoning. Akular et al. [38] developed language-guided adaptive convolutional layers, allowing the model to co-attend to salient features within visual and textual information.

Recently, graph neural networks (GNNs) have gained traction in VQA because of their effectiveness in capturing both global and local relationships. Teney et al. [39] used GNNs to represent relationships among scene objects and question words, inspiring Brown et al. [40] to propose a graph-based VQA approach incorporating a graph learner module that captures image representations specific to the question. Hu et al. [41] proposed a language-conditioned graph network to capture relationships among objects and between objects and language. Khademi et al. [42] introduced a multimodal neural graph memory network for VQA. Hudson et al. [43] enhanced model interpretability by incorporating a neural state machine that predicts a semantic relation probability graph for the image, performing sequential reasoning on the probability graph to evaluate effectiveness.

### 2.4 Existing issues with current methods

VQA requires simultaneous understanding of visual and textual content, with the fusion of these multimodal features for effective answer generation. Efficient VQA model performance relies on accurate text and image feature extraction and effective multimodal feature fusion. However, current VQA methods face several challenges: (1) Existing VQA models often only capture global visual features, neglecting crucial local details, which are often necessary for accurate question answering. (2) Insufficient interaction between visual and textual modalities. Although attention mechanisms are employed, they generally prioritize image regions guided by questions, lacking reciprocal guidance of text by image. Additionally, interference control between features during fusion remains underexplored. (3) Most VQA approaches operate as classification tasks (selecting the correct answer from a set of options), which falls short in real-world applications where generative answers are more applicable. Moreover, there is no standard evaluation mechanism for generated answers. (4) Limited model interpretability. Current VQA methods do not explain the reasoning behind answer selection. An interpretable network design is needed to provide justifications alongside answer predictions.

## 3. The proposed LGMTNet

**Fig 1:**
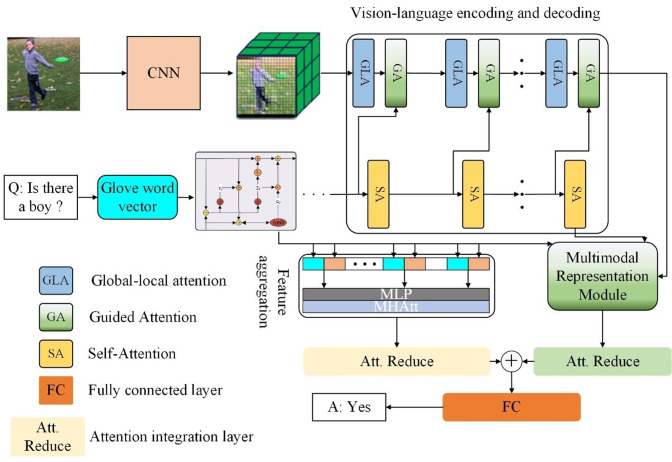
The structure of LGMTNet.

To overcome the shortcomings of the Transformer’s self-attention mechanism in distilling local details and insufficient multimodal feature fusion, a GLA and dual fusion strategy for visual-language features are proposed. This approach emphasizes key local features within global image representations while enhancing multimodal integration, thereby improving model performance. The proposed method is structured into three main components: an advanced vision-language encoding and decoding framework and decoding module, a multimodal feature representation learning module, and a question-feature and multimodal feature aggregation module. The structure of LGMTNet is presented in **Fig 1**. The visual feature extraction module employs ResNet-101 to extract image features, with an input size of 224 × 224 and an output feature map of dimension ${\mathbb{R}}^{2048\times 7\times 7}$ . Global average pooling is then applied to obtain the final visual feature representation ${\mathbb{R}}^{2048}$. The textual feature extraction module utilizes a gated recurrent unit (GRU) to process the input text sequence, with an embedding dimension of 300 and a hidden state size of 512, resulting in a final text feature dimension of ${\mathbb{R}}^{512}$. The multi-layer perceptron (MLP) has an input dimension of ${\mathbb{R}}^{\left(2048+512\right)}$ and an output dimension of ${\mathbb{R}}^{1024}$. The multi-head attention (MHAtt) module takes an input of dimension ${\mathbb{R}}^{1024}$ and produces an output of dimension ${\mathbb{R}}^{1024}$.

### 3.1 Deep vision-language encoding and decoding framework

#### 3.1.1 Global-local l attention.

**Fig 2:**
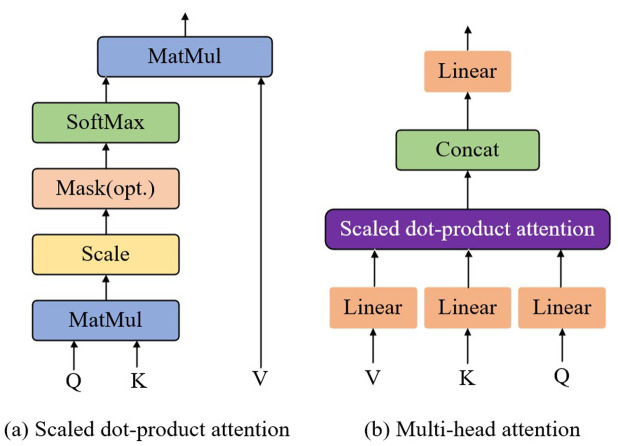
Structures of scaled dot-product attention and multi-head attention.


As a fundamental component of self-attention, multi-head attention plays a crucial role in modeling feature relationships, as illustrated in **Fig 2(b). One of its key components, the scaled dot-product attention, is shown in Fig 2(a)**. In multi-head attention, this mechanism is stacked *h* times. The modeling process can be understood as follows: the input text features $T\in {\mathbb{R}}^{n\times d}$ (where *n* denotes the number of words and *d* represents the dimensionality) are linearly mapped to obtain the queries *Q*, keys *K*, and values *V*. The dot product of *Q* and *K* is then computed and scaled by a factor of $\sqrt{d}$, after which the attention scores are derived using the softmax function. Finally, the attention features $f_{att}$ are obtained by multiplying the result with *V*. To simplify calculations and reduce computational complexity, the dimensions of *Q*, *K*, and *V* are kept consistent. The calculation process is shown in Eq. (1).

$$f_{att}=Att(Q,K,V)=softmax\left(\frac{QK^{T}}{\sqrt{d}}\right)V$$ (1)

To further enhance the representational power of the attention features, multiple independent attention heads $f_{att}$ are concatenated. Consequently, the final multi-head attention output is given by:


$$\begin{array}{cc} & F=MHAtt(Q,K,V)=\left[f_{att_{1}},f_{att_{2}},\cdots ,f_{att_{h}}\right]W^{o}\\ & f_{att_{i}}=Att(QW_{i}^{Q},KW_{i}^{K},VW_{i}^{V}) \end{array}$$ (2)

where $W_{i}^{Q},W_{i}^{K},W_{i}^{V}\in {\mathbb{R}}^{d\times d_{h}}$ represents the mapping matrix for each head, and $W^{o}\in {\mathbb{R}}^{h*d\times d_{h}}$ is learnable. The notation [⋅] denotes concatenation. Here, *F* represents the features obtained from the multi-head attention calculation, *h* denotes the number of heads in the multi-head attention, $d_{h}$ represents the dimensionality of the output features for each head, and *d* is the size of the feature vectors. To prevent an excessive number of parameters in the multi-head attention mechanism, it is agreed that $d_{h}=d/h$.

**Fig 3:**
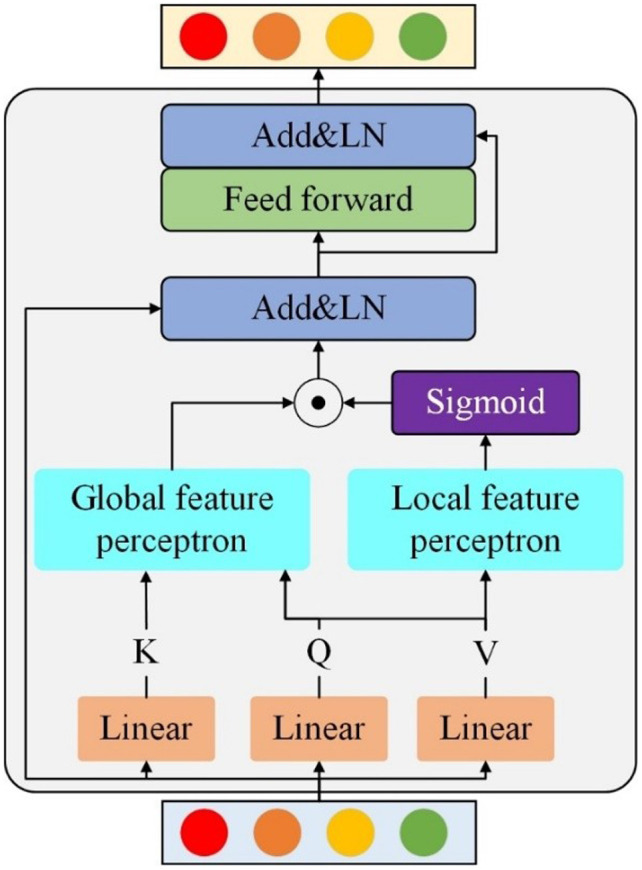
Structure of global-local attention.


To enhance the model’s ability to accurately understand complex input data, this study introduces a hybrid attention mechanism that incorporates both global and local attention. First, the multi-head attention mechanism described above is employed to model global feature relationships and extract global features. Next, a local attention mechanism with path selection capabilities is used to extract key local features. Finally, the local key features are utilized to generate a relevant weight, which is applied to the global features extracted by the multi-head attention. This process effectively prioritizes attention on the local key features. The proposed global-local attention (GLA) is illustrated in **Fig 3**. The global feature perceptron (corresponding to Eq. (4)) is responsible for capturing long-range dependencies, while the local feature perceptron (corresponding to Eq. (5)) employs a dynamic attention mechanism to extract key local features. These components work synergistically to enhance feature representation. The global feature perceiver represents the self-attention mechanism with residual connections and layer normalization (LN) removed, while the local feature perceiver corresponds to a dynamic attention layer $DSA_{R}$ designed for extracting key visual features. Its expression is as follows:

$$DSA_{R}\left(X\right)=soft\max\left(\frac{XW_{q}{\left(XW_{k}\right)}^{T}}{\sqrt{d}}\sum {\mathrm{\backslash nolimits}}_{i=0}^{n}\lambda_{i}M_{i}\right)XW_{v}$$ (3)

where $W_{q}$, $W_{k}$ and $W_{v}$ represent learnable weight parameters, which are shared across different layers to reduce the overall number of parameters. $\lambda_{i}$ denotes the weight coefficient, which adjusts the importance of different features, while $M_{i}$ represents the attention mask matrix, used for local feature extraction and dynamic attention computation.

Subsequently, the features are fed into the multi-head attention mechanism by distilling the global features, referred to as $V^{\prime }$.


$$V^{\prime }=MHAtt\left(Q,K,V_{1}\right)$$ (4)

where $V_{1}$ represents the “value” matrix in the first layer of the GLA module. Additionally, it should be noted that Eq. (4) describes the computation process of a single GLA layer.

Key local features, represented as $L^{\prime }$, generate a weight matrix that is subsequently applied through element-wise multiplication with the global features $V^{\prime }$. This operation results in the extraction of the key local features.


$$F_{local}=V^{\prime }⊙\sigma \left(L^{\prime }\right)$$ (5)

where ⊙ represents the element-wise Hadamard product, and σ denotes the sigmoid activation function. The extracted key local features, denoted as $L^{\prime }$ ′, are obtained by applying the generated weight matrix through element-wise multiplication with the global features. This process enhances the most relevant local details while preserving crucial global contextual information, thereby improving feature representation. The resulting local key features $F_{local}$ are combined with the input features *X* through a residual connection and then passed through a feedforward network (FF) and LN to obtain more expressive features $F_{local}^{\prime }$. The FF network consists of two fully connected layers (FC), an activation function (GELU), and dropout. The entire process is represented as follows:


$$\begin{array}{cc} & F_{local}^{\prime }=LN\left(f+FF\left(f\right)\right)\\ & \;f=LN\left(X+F_{local}\right) \end{array}$$ (6)

where *f* represents the features obtained after normalizing the local relational features and the input features.

#### 3.1.2 Co-attention layer.

**Fig 4:**
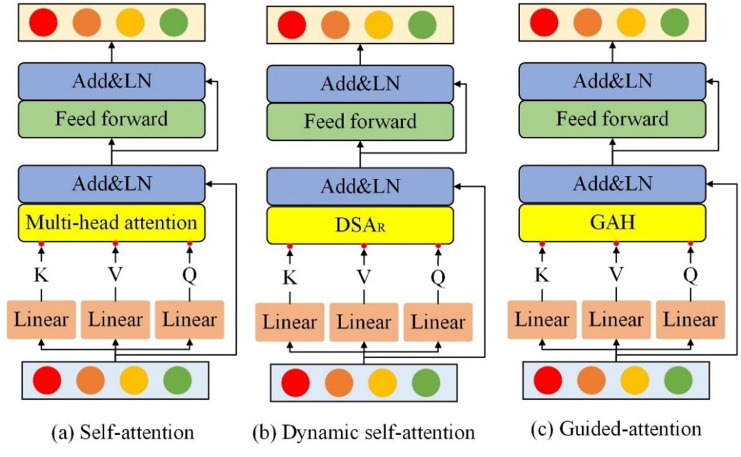
Structures of the three basic attention units.


The co-attention Layer (CAL) consists of three basic attention units: self-attention (SA), GLA, and guided-attention (GA). The structure of GLA is shown in **Fig 3**, while the structures of SA, dynamic self-attention (DSA), and GA are depicted in **Fig 4**. SA is designed based on multi-head attention, while DSA is based on the local feature perceiver proposed in this study. The guided attention head (GAH) modifies the traditional multi-head attention by incorporating two modal inputs: text $T\in {\mathbb{R}}^{n\times d}$ and image $X\in {\mathbb{R}}^{m\times d}$, instead of the single-modal input used in MHAtt. The calculation process is represented as follows:

$$\begin{array}{cc} & f_{t}=GAH(T,X,X)=\left(\frac{TW_{q}{\left(XW_{k}\right)}^{T}}{\sqrt{d}}\right)XW_{v}\\ & {\tilde{f}}_{t}=LN(T+f_{t})\\ & F_{GA_{t}}=LN({\tilde{f}}_{t}+FF(\tilde{f})) \end{array}$$ (7)

**Fig 5:**
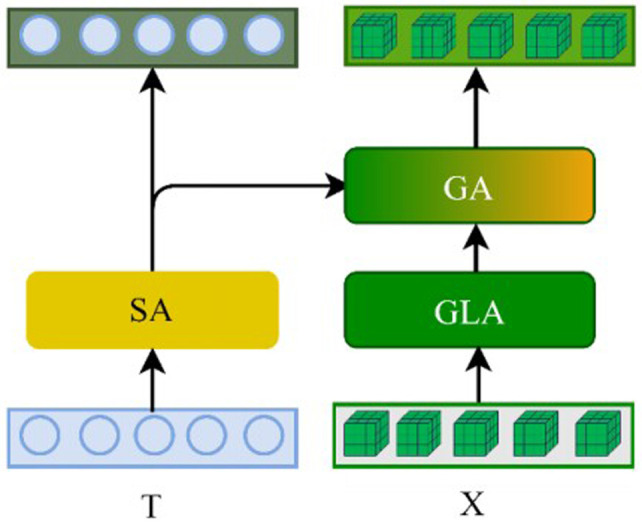
Structure of CAL network.

Fig 5 illustrates a basic CAL unit, where T and X represent the question features and visual features, respectively. In the co-attention layer, GLA and SA are first applied to encode the visual and textual features separately, capturing intra-modal relationships. The encoded features are then fed into the GA unit, where the textual features act as a ‘guide’, directing the model to understand the natural language description of the visual elements, thus enhancing information fusion between the visual and language modalities.

#### 3.1.3 Deep vision-language encoding and decoding framework.

**Fig 6:**
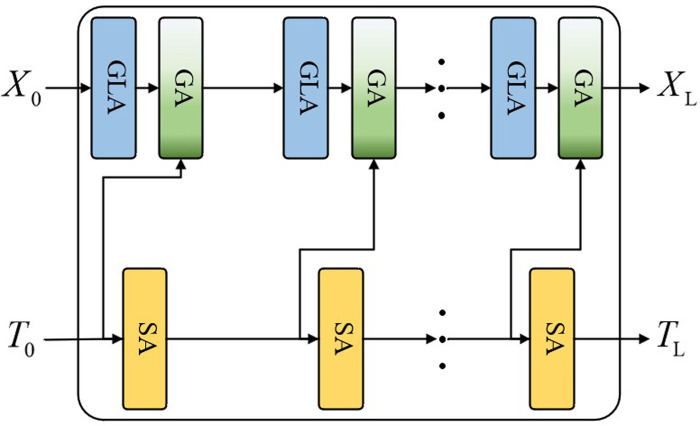
Structure of D-VLEDF network.

As shown in **Fig 6**, the output of SA after encoding the textual features can be passed to GA for modal fusion or directly to the next layer. This design enables flexibility in controlling the number of CAL units within the deep vision-language encoding and decoding framework (D-VLEDF). Specifically, multiple CAL units are assembled following the encoder-decoder framework of a Transformer, with the structure illustrated in **Fig 6**.

In this deep encoder-decoder framework, visual features X and question features T are fed into a series of CAL units (denoted as $CAL^{1}$, $CAL^{2}$,..., $CAL^{L}$) for deep co-attention learning. The input features for $CAL^{L}$ are represented by $X_{L-1}$ and $T_{L-1}$, thus allowing the encoding-decoding process in D-VLEDF to be expressed as follows:


$$X_{L},T_{L}=CAL^{L}\left(X_{L-1},T_{L-1}\right)$$ (8)

where $X_{L}$ and $T_{L}$ represent the output features of the $L_{th}$ CAL layer, which can then be passed to $CAL^{\left(L+1\right)}$, setting the input features of $CAL^{1}$ to $X_{0}=X$ and $T_{0}=T$.

### 3.2 Multimodal feature representation module

**Fig 7:**
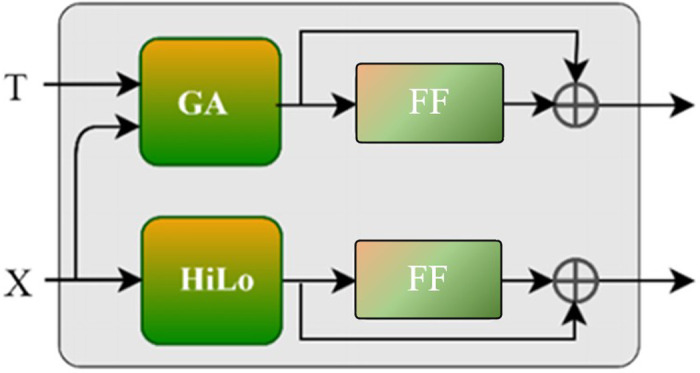
Structure of multimodal feature representation module.

This module focuses on GA and high-low frequency attention (HiLo). In the deep encoder-decoder module, GA leverages question features to guide the learning of visual features, effectively instructing the model on “what to see” by capturing visual features relevant to the question terms. However, for accurate prediction, a VQA model must also understand “what to hear.” Therefore, an additional GA unit is incorporated in the multimodal representation module (MMR), reinforcing the understanding of “what to hear” and further strengthening the visual-language feature integration. Moreover, high-low frequency attention is introduced to improve comprehension of the encoded multimodal features containing question information. The overall structure of MMR is depicted in **Fig 7**.

The feature representation process in this module can be formulated as follows:


$$\begin{array}{cc} & f_{T}=f_{t}+FF\left(f_{t}\right)\\ & f_{t}=GMHAtt\left(T,X,X\right)=soft\max\left(\frac{TW_{q}{\left(XW_{k}\right)}^{T}}{\sqrt{d}}\right)XW_{v} \end{array}$$ (9)

$$\begin{array}{cc} & f_{X}=f_{x}+FF\left(f_{x}\right)\\ & f_{x}=HiLo\left(X\right) \end{array}$$ (10)

where $f_{t}$ represents the attention results after computing image-guided question attention, and $f_{T}$ represents the enhanced question features after residual connection and FF. To further improve visual-language fusion, additional GA layers can be introduced as needed. $f_{x}$ represents the visual features obtained from HiLo, and $f_{X}$ is computed in the same way as $f_{T}$.

#### 3.2.1 High and low frequency attention mechanism.

Essentially, the low-frequency attention branch (Lo-Fi) is designed to capture broad feature dependencies, leveraging global attention without requiring high-resolution feature maps. Conversely, the high-frequency attention branch (Hi-Fi) captures detailed local dependencies, which necessitates high-resolution feature maps and operates effectively with local attention. Each of these attention types is discussed in detail below.

Hi-Fi primarily encodes fine-grained local details. Applying global attention to the feature map could inadvertently connect unrelated objects, hindering model optimization. Hence, Hi-Fi is designed to capture detailed features within local windows, thereby reducing computational complexity. Unlike time-intensive operations such as window-shifting or multi-scale window partitioning, Hi-Fi utilizes a non-overlapping window partitioning approach, which is more hardware-friendly.

Recent studies have demonstrated that global attention in MHAtt can effectively capture low-frequency information. However, directly applying MHAtt to high-resolution feature maps incurs substantial computational costs. In low-frequency attention (Lo-Fi), average pooling—acting as a low-pass filter—is first applied across each channel to extract low-frequency signals from the input features. These pooled feature maps are then mapped into keys $K\in {\mathbb{R}}^{n/s^{2}\times d}$ and values $V\in {\mathbb{R}}^{n/s^{2}\times d}$, with *s* representing the window size. Queries *Q* in Lo-Fi are derived from the original feature map, allowing standard attention to capture comprehensive low-frequency information across the feature map. With reduced spatial dimensions for keys *K* and values *V*, Lo-Fi significantly lowers the computational complexity of MHAtt.


$$f_{att}=Att\left(Q,K,V\right)=soft\max\left(\frac{QK^{T}}{\sqrt{d}}\right)V$$ (11)

$$F=MHAtt\left(Q,K,V\right)=[f_{att_{1}},f_{att_{2}},\cdots ,f_{att_{h}}]W^{o}$$ (12)

The initial allocation of heads in Hi-Fi and Lo-Fi follows the original number used in MHAtt. However, excessive head counts may increase computational costs. To optimize efficiency, the heads from MHAtt are split into two groups with a defined ratio α; (1−α)h heads are allocated to Hi-Fi, while the remaining αh heads are assigned to Lo-Fi. This split reduces the computational load of HiLo, as both attention mechanisms are less complex than standard MHAtt. An additional benefit of this head-splitting strategy is the reduction of model parameters by decomposing the learnable parameter matrix $W^{o}$ in Eq. (12) into two smaller matrices. Finally, the results from Hi-Fi and Lo-Fi are concatenated to form the final attention output, denoted by:


$$f_{HiLo}=HiLo\left(X\right)=\left[HiFi\left(X\right)\|LoFi\left(X\right)\right]$$ (13)

where [·‖·] represents the concatenation of these two types of features.

#### 3.2.2 Multimodal feature representation.

As shown in **Fig 7**. unlike the GA in the deep encoder-decoder module, this GA utilizes visual features to guide question feature learning (Eq. (9)), thereby enhancing the fusion between visual and linguistic features and reducing language noise in the question. Additionally, high and low frequency attention processes the global and local information within visual features to capture critical high- and low-frequency elements from the multimodal features, thereby enhancing the representation capability of the visual features. Subsequent experiments validating the effectiveness of the multimodal feature representation module indicate that it improves the representation capacity of multimodal features and thus enhances answer prediction accuracy.

### 3.3 Feature aggregation module

**Fig 8:**
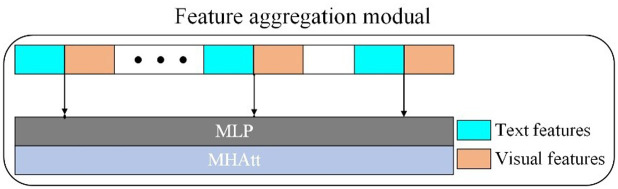
Structure of the feature aggregation module.

This module is designed to compute the relationship between textual features and the visual-language bimodal embedding. The bimodal embedding captures the interactions between textual and visual features. Therefore, the module aims to effectively capture the association between the query and the bimodal representation, enabling the retrieval of the most relevant information from the bimodal features. The structure of the feature aggregation module is illustrated in **Fig 8**.

This module first concatenates the question features *Q* output by the GRU with the multimodal features $f_{x}$ processed by the deep encoder-decoder and multimodal representation module:


$$F_{concat}=\text{Concat}[Q;f_{x}]$$ (14)

where $F_{concat}$ represents the concatenated features.

The concatenated features are processed through adaptive average pooling (AAP) and a MLP to ensure that the final feature dimensions align with those of the textual features while enhancing the understanding of the fused representation. The “adaptive” nature of AAP refers to dynamically adjusting the pooling window size and stride based on the input feature dimensions, ensuring that the output features match the required textual feature dimensions, as defined in Eq. (15).


$$F_{pool}=AAP(F_{concat})$$ (15)

Subsequently, the MLP further transforms the feature representation, as defined in Eq. (16).


$$F_{mlp}=MLP(F_{pool})$$ (16)

After obtaining the standardized features, MHAtt is applied to model the concatenated features, further enhancing the fusion of the two modalities, as shown in Eq. (17).


$$F_{att}=MHAtt(F_{mlp})$$ (17)

To enhance the representational capability of MHAtt, a feedforward network (FF) is introduced in practical applications. This network consists of two fully connected (FC) layers, a ReLU activation function, and dropout regularization, as defined in Eq. (18).


$$F_{final}=FF(F_{att})=FC(ReLU(Dropout(F_{att})))$$ (18)

The multimodal features undergo multiple layers of computation, thoroughly integrating rich semantic information. This enables the VQA model to more accurately understand the question semantics and enhances its ability to predict the correct answer.

## 4. Experiments and conclusion analysis

### 4.1 Experimental datasets and evaluation metrics

#### 4.1.1 Dataset.

VQA has been a research focus for several years, resulting in the development of numerous datasets. These datasets have grown significantly in size and contain increasingly comprehensive annotations. The commonly used datasets in VQA research include COCO-QA, VQA-1.0, VQA-2.0, and Visual Genome. The experiments are designed with the VQA-2.0 dataset.

The VQA-2.0 dataset is an extension of the VQA-1.0-real dataset, specifically designed to mitigate language biases inherent in VQA-1.0-real. By addressing these biases, VQA-2.0 prevents models from relying on language priors to achieve higher prediction accuracy, facilitating the development of more interpretable VQA models that focus on visual content. Specifically, the VQA-2.0 dataset includes 82,783 images with 443,757 questions for training, 40,504 images with 214,354 questions for validation, and 81,434 images with 447,793 questions for testing. This dataset is approximately double the size of VQA-1.0 and is divided into three categories: “Yes/No,” “Number,” and “Other.” Additionally, each question corresponds to two visually similar yet distinct images, enabling the model to generate different answers. This dual correspondence effectively reduces bias and imbalance in the dataset.

#### 4.1.2 Evaluation metrics.

VQA models are trained to automatically answer questions based on visual and linguistic information. Evaluation metrics primarily assess whether the predicted output matches the correct answer, with Accuracy as the primary metric.

Ren et al. [44] framed VQA as a classification task, using Accuracy (*Acc*) as the standard performance metric for VQA models. However, in practical applications, a question may have multiple correct answers. To account for this, Antol et al. [45] provided multiple human-annotated answers for each question. In this study, the experiments rely on the VQA-2.0 dataset, using Accuracy as the evaluation metric. Higher values indicate better model prediction accuracy. The calculation of the accuracy of a predicted answer *a* is presented in:


$$Acc\left(a\right)=\min\left(1,\frac{C_{a}}{3}\right)$$ (19)

where $C_{a}$ represents the number of occurrences of the predicted answer in the set of human-annotated answers. If three or more annotations agree on the same answer, *a* is considered correct.

### 4.2 Experimental environment and network parameters

Hardware requirements: This study is conducted on a server running Ubuntu 20.04.4 LTS, with the following specifications: CPU – Intel® Xeon® E5-2650 V4 @ 2.20GHz × 48, RAM – 128GB, GPU – NVIDIA TITAN V with 12GB memory. To enhance training efficiency, two GPUs are utilized.

Software requirements: Python 3.7, PyTorch 1.7, and CUDA 11.7 are used. PyTorch, a Python-based scientific computing library, enables flexible model adjustments and optimizations through dynamic computational graphs. With built-in optimizers like SGD, Adam, and Adagrad, PyTorch facilitates the implementation of various optimization algorithms.

Experimental parameter settings: Image features are extracted using ResNext152, pre-trained on Visual Genome, yielding visual features with dimensions of 256 × 2048 after max-pooling, further mapped to 256 × 512 for computational convenience. For textual features, question lengths are set to 16, with 300-dimensional GLoVe embeddings processed through a GRU to obtain language features of 16 × 512 dimensions. The attention module employed 8 heads, each with a dimension of 64. During training, the batch size is 64, and the learning rate is updated per the function $\min\left(2.5te^{-5},1e^{-4}\right)$, where *t* represents the current epoch, s*t*arting from 1. After 10 epochs, the learning rate is reduced by a factor of 5 every two epochs. Adam served as the optimizer, with the model trained for 13 epochs in total.

### 4.3 Comparison with other methods

BAN [9] employs a bilinear attention network to extract bilinear interactions between image regions and question words, generating joint representations through low-rank bilinear pooling. DFAF [31] alternates intra- and inter-modality interactions across visual and linguistic modalities, improving predictive performance. ReGAT [46] uses graph attention to model interactions among multiple object types within each image, learning adaptive relations for questions and target objects, thus improving prediction accuracy. MCAN [32] captures self-modal and image-guided attention through deep co-attention modules, improving answer prediction accuracy. MMnasNet [47] constructs a deep encoder-decoder backbone with a task head for VQA, enhancing answer accuracy. Re-Att [48] refocuses visual information in images based on answer information, achieving strong performance. APN [49] introduces an automatic parsing network that identifies and leverages latent tree structures in data, enhancing Transformer-based vision-language systems’ efficacy in VQA and image captioning. ARAC [50] proposes a fully attention-based VQA architecture, including an answer-checking module that applies unified attention to common answers, questions, and image representations, updating the answer. MCAoAN [51] develops an attention module (AoA) to clarify relationships between attention maps and query sets, yielding a final feature matrix by multiplying an attention weight matrix with the attention map.

**Table 1** shows that the proposed LGMTNet outperforms other algorithms on the VQA-2.0 test set, achieving overall accuracies of 72.04% and 72.29% across two test sets. Compared with MCAN, LGMTNet improves Test-dev accuracy by 1.41% and Test-std by 1.39%, showing respective gains of 1.28% and 2.07% in Number and Other question types. Significant improvements are also observed over ReGAT, with LGMTNet’s overall accuracy surpassing it by 1.77% and 1.71% on the two test sets. These findings confirm that the proposed global-local relationship attention mechanism enhances the model’s focus on local salient features, supporting more accurate answer prediction and improving VQA performance.

**Table 1:** Accuracy comparison with other algorithms on the VQA-2.0 test set.

Method	Test-dev				Test-std
	All	Yes/No	Number	Other	All
BAN	70.04	84.48	54.04	60.52	70.35
ARAC	70.06	85.90	54.04	60.52	70.40
DFAF	70.22	86.09	53.32	60.49	70.34
ReGAT	70.27	86.08	54.42	60.33	70.58
Re-Att	70.43	87.00	53.06	60.19	70.72
MCAN	70.63	86.82	53.26	60.72	70.90
MCAoAN	70.90	87.05	53.81	60.97	71.14
APN	71.14	87.44	52.68	61.18	71.33
MMnasNet	71.24	87.27	55.68	61.05	71.46
Proposed	72.04	87.52	54.54	62.79	72.29

**Fig 9:**
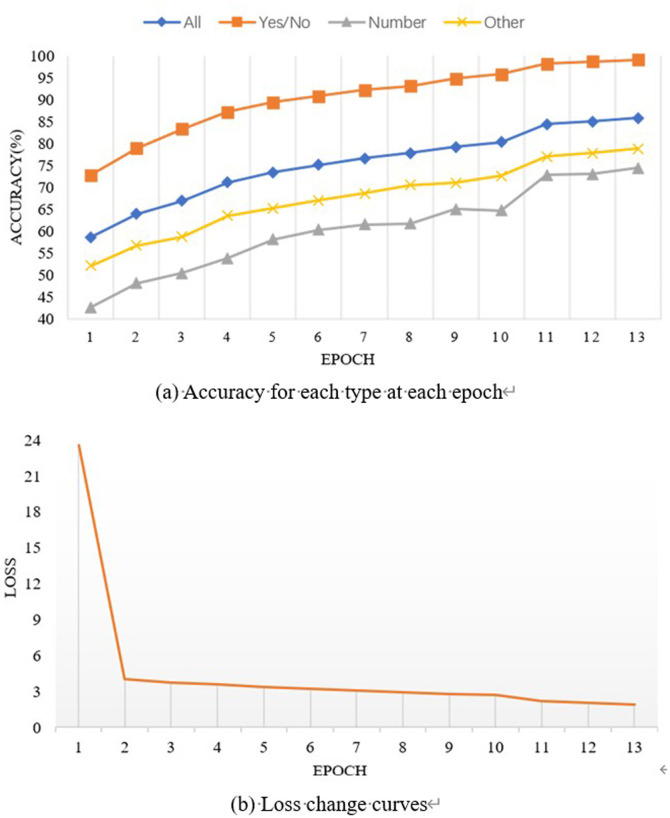
Accuracy and loss curves by epoch for each question type.

**Fig 9(a)** shows LGMTNet’s training progress on the VQA-2.0 dataset, involving training, validation, and VisualGenome datasets, with validation on the validation set. Although the results exceed those in Table 6, optimal accuracies across question types are reached by epoch 13. **Fig 9(b)** shows overall loss decreasing with each training iteration, stabilizing at epoch 13, indicating that the model had fully learned.

**Fig 10:**
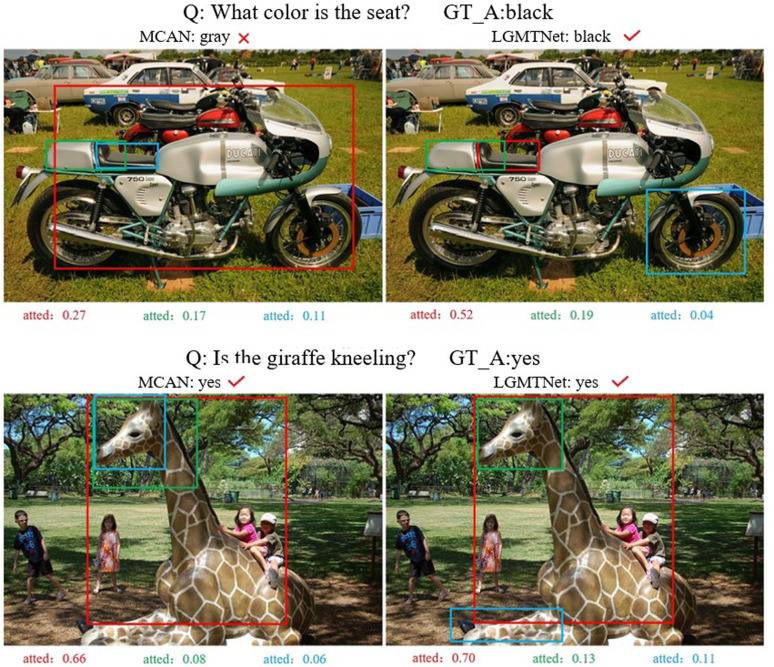
Visualization of MCAN and LGMTNet on VQA-2.0.

The visual results of the proposed LGMTNet method and the baseline model MCAN on selected examples from the VQA-2.0 dataset, along with the corresponding attention weights on the visual regions, are shown in **Fig 10**. For the question “What color is the set?”, correctly answering this question requires the model to learn position-related information. As seen in the figure, LGMTNet, with the aid of DSA, is able to more accurately locate the “set” and provide the correct answer (highlighted in red). In the question “Is the giraffe kneeling?”, both models answer the question correctly, but the proposed model is able to capture more fine-grained feature information through the attention mechanism, enabling it to reason more accurately. The visualization shows that, after using DSA, the model can focus on information related to the “giraffe’s” legs and their interaction with the ground (highlighted in blue). These visualizations further demonstrate that dynamic attention enhances the model’s ability to capture implicit relationships between different modalities, thereby facilitating a deeper understanding of the high-level semantics of the image and the question, and improving the model’s overall performance.

### 4.4 Ablation study

#### 4.4.1 Comparison of GLA with SA and DSA.

To validate the effectiveness of the GLA mechanism, this study compares the proposed method with SA and dynamic self-attention (DSA), and the details are shown in **Table 2**.

**Table 2:** Comparison results.

Method	val			
	All	Yes/No	Number	Other
SA	68.42	85.53	51.20	59.96
DSA	68.45	85.24	51.42	60.19
GLA	68.75	85.70	51.68	60.36

To accelerate experimentation, these tests are conducted with a reduced encoder-decoder depth (L = 2) and without the multimodal feature representation or feature aggregation modules. The GLA algorithm successfully identifies question-relevant local features within global contexts. Compared with SA, GLA achieves notably higher accuracy for “Number” and “Other” question types, with improvements of 0.48% and 0.40%, respectively. Since GLA captures key local features within global image features, it proves advantageous for question types that require attention to localized visual objects. For instance, when answering “What is the lady holding in her hand?”, the model needs to focus on the local detail “coffee pot” to answer accurately. The observed accuracy improvements in these question types highlight GLA’s superior capability in local feature detection compared to SA and DSA, especially for the “Other” category where similar properties exist.

Based on the experimental results in **Table 2**, a paired *t*-test is conducted to perform statistical significance analysis on the results. The differences between GLA and SA/DSA across four categories are compared by calculating the corresponding *p*-values to determine whether the differences are statistically significant. The steps for the paired *t*-test between GLA and SA are as follows. The mean difference between GLA and SA across the four metrics—All, Yes/No, Number, and Other—is $\bar{d}=0.355$, with a standard deviation of $s_{d}=0.140$. The t-value is then calculated using the formula $t=\frac{\bar{d}}{s_{d}/\sqrt{n}\;}$, with n representing the sample size, resulting in a t-value of 5.07. Referring to the *t*-distribution table, the *p*-value is found to be p < 0.01, indicating that the accuracy difference between GLA and SA is statistically significant. Similarly, the *t*-value between GLA and DSA is calculated to be 4.47, and the *p*-value obtained from the *t*-distribution table is p < 0.01, confirming that the accuracy difference between GLA and DSA is statistically significant. The results of the paired *t*-test fur*t*her validate that GLA demonstrates statistically significant accuracy improvements over both SA and DSA across all categories. Although the differences in some ca*t*egories are relatively small, GLA still shows an advantage in overall accuracy, particularly in capturing local visual features.

**Fig 11:**
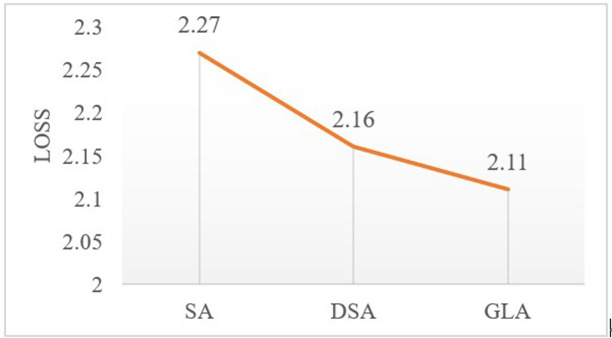
Loss comparison among SA, DSA, and GLA.

A comparison is conducted between the final epoch loss values after convergence for the three approaches. The values are illustrated in **Fig 11**, indicating that the model incorporating the GLA method demonstrates superior convergence and achieves higher predictive accuracy.

#### 4.4.2 Analysis of CAL module variants.

**Fig 12:**
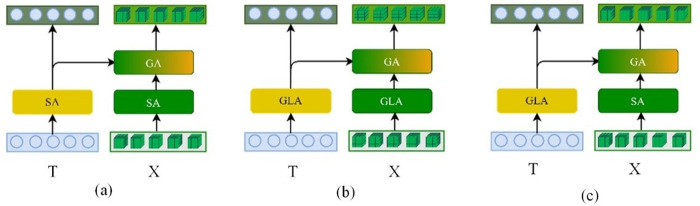
Variants of CAL module. (a) SA, (b) GLA, (c) GA.

Variants of CAL module is illustrated in **Fig 12**. To investigate optimal structures for the D-VLEDF in enhancing VQA model performance, several CAL module variants are designed and experimentally tested, with results detailed in **Table 3**. The SA (T)-SGA (X, T) design from Ref [25] is used as the baseline for verification. **Table 3** shows that adding GLA to the baseline model improves overall model accuracy. Notably, when GLA is applied to both visual and question features, the model shows a marked improvement in handling Number-related questions. This suggests that integrating spatial position aids in resolving counting-related queries. However, compared to SA(T)-GGLA (X, T), applying GLA to both features introduces noise (e.g., articles, prepositions) that affects performance in other categories. Thus, the SA(T)-GGLA (X, T) structure, which optimally balances whole identification results, is selected for building the D-VLEDF.

**Table 3:** Performance comparison of CAL module variants.

Method	val			
	All	Yes/No	Number	Other
SA (T)-SGA (X, T)	67.31	84.91	49.41	58.71
GLA (T)-GGLA (X, T)	67.78	85.35	52.13	59.23
GLA (T)-SGA (X, T)	67.76	85.28	49.26	59.38
SA (T)-GGLA (X, T)	68.75	85.70	51.68	60.36

Based on the experimental results in **Table 3**, a paired t-test is conducted to perform statistical significance analysis on the results. The differences between SA (T)-GGLA (X, T) and SA (T)-SGA (X, T), GLA (T)-GGLA (X, T), and GLA (T)-SGA (X, T) across four categories are compared by calculating the corresponding p-values to determine whether the differences are statistically significant.

The *t*-value between SA (T)-GGLA (X, T) and SA (T)-SGA (X, T) is 4.11, with a corresponding p-value of 0.016. Since the p-value is less than 0.05, this indicates that the accuracy difference between SA (T)-GGLA (X, T) and SA(T)-SGA (X, T) is statistically significant. The t-value between SA (T)-GGLA (X, T) and GLA(T)-GGLA (X, T) is 1.85, with a p-value of 0.14. Since the p-value is greater than 0.05, it suggests that the accuracy difference between SA (T)-GGLA (X, T) and GLA (T)-GGLA (X, T) is not statistically significant. Finally, the t-value between SA (T)-GGLA (X, T) and GLA (T)-SGA (X, T) is 2.54, with a p-value of 0.046. Since the p-value is less than 0.05, this indicates that the accuracy difference between SA (T)-GGLA (X, T) and GLA (T)-SGA (X, T) is statistically significant.

#### 4.4.3 Impact of encoder-decoder layer count on experimental accuracy.

**Fig 13:**
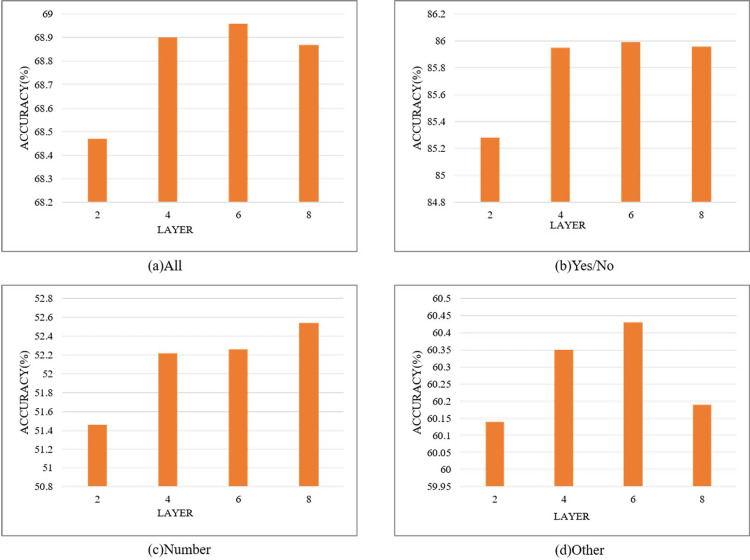
Accuracy for different layer count encoder-decoder configurations.

To verify the effect of the encoder-decoder layer count on model performance, multiple values of L∈{2,4,6,8} are tested. Detailed experimental results are shown in **Fig 13**. **Fig 13(a)** illustrates that overall and individual type accuracy increase with the number of layers until Layer 6. After exceeding six layers, accuracy declines, establishing Layer 6 as optimal for constructing the deep encoder-decoder.

Based on the experimental results in **Fig 13**, a paired *t*-test is conducted to perform statistical significance analysis on the results. The differences in accuracy between 6 layers and 2/4/8 layers are compared by calculating the corresponding p-values to determine whether the differences are statistically significant.

The *t*-value between 6 layers and 2 layers is 4.79, with a corresponding p-value of 0.008. Since the *p*-value is less than 0.05, this indicates that the accuracy difference between 6 layers and 2 layers is statistically significant. The *t*-value between 6 layers and 4 layers is 6.00, with a *p*-value of 0.006. Since the p-value is less than 0.05, this suggests that the accuracy difference between 6 layers and 4 layers is statistically significant. The *t*-value between 6 layers and 8 layers is 0.20, with a *p*-value of 0.85. Since the *p*-value is greater than 0.05, the accuracy difference between 6 layers and 8 layers is not statistically significant. The statistical analysis results indicate that the accuracy differences between 6 layers and 2 layers, as well as between 6 layers and 4 layers, are significant, while the difference between 6 layers and 8 layers is not significant.

#### 4.4.4 Effectiveness verification of multimodal representation and feature aggregation modules.

To enhance multimodal feature representation, a multimodal representation (MMR) module and a feature aggregation (Agg) module are incorporated following the deep encoder-decoder, aiming to improve VQA model performance. The experiment compared the D-VLEDF with the module combined with MMR (D-VLEDF+MMR), Agg (D-VLEDF+Agg), and both (D-VLEDF+MMR + Agg) using the VQA-2.0 dataset. Results are shown in **Table 4**.

**Table 4:** Validation of module effectiveness.

Method	val			
	All	Yes/No	Number	Other
D-VLEDF	68.79	86.16	51.32	60.20
D-VLEDF+MMR	68.96	85.86	52.39	60.49
D-VLEDF+Agg	68.93	86.08	52.23	60.31
D-VLEDF+MMR + Agg	68.96	85.99	52.26	60.43

**Table 4** shows that adding the MMR module improved overall, Number, and Other accuracy by 0.17%, 1.07%, and 0.29%, respectively, highlighting MMR’s enhancement of multimodal feature representation and its particular benefit in answering Number-related questions. Adding the Agg module further improved accuracy by 0.14%, 0.91%, and 0.11% for these classes, indicating the beneficial role of the aggregation process on model performance. Both MMR and Agg utilize multi-step fusion strategies that effectively enhance model performance. Compared to the Agg module, MMR produced a more notable improvement, underscoring the importance of simultaneously understanding visual and auditory inputs in VQA. In contrast, Agg’s simple feature concatenation proved insufficiently refined, limiting performance gains. However, using MMR and Agg together yielded accuracy improvements across question types, affirming the effectiveness of both modules in enhancing prediction precision.

Based on the experimental results in **Table 4**, a paired t-test is conducted to perform statistical significance analysis on the results. The differences in accuracy between D-VLEDM+MMR + Agg and other models (D-VLEDM, D-VLEDM+MMR, D-VLEDM+Agg) are compared by calculating the corresponding p-values to determine whether the differences are statistically significant. The *t*-value between D-VLEDM+MMR + Agg and D-VLEDM is 1.21, with a corresponding *p*-value of 0.29. Since the p-value is greater than 0.05, this indicates that the accuracy difference between D-VLEDM+MMR + Agg and D-VLEDM is not statistically significant. The *t*-value between D-VLEDM+MMR + Agg and D-VLEDM+MMR is -0.30, with a *p*-value of 0.79. Since the *p*-value is greater than 0.05, the accuracy difference between D-VLEDM+MMR + Agg and D-VLEDM+MMR is not statistically significant. The *t*-value between D-VLEDM+MMR + Agg and D-VLEDM+Agg is 0.36, with a *p*-value of 0.75. Since the *p*-value is greater than 0.05, this suggests that the accuracy difference between D-VLEDM+MMR + Agg and D-VLEDM+Agg is not statistically significant. The statistical analysis results indicate that the accuracy differences between D-VLEDM+MMR + Agg and the other model variants (D-VLEDM, D-VLEDM+MMR, D-VLEDM+Agg) are not significant. Although D-VLEDM+MMR + Agg shows slight improvements in some categories, these differences do not reach statistical significance.

**Fig 14:**
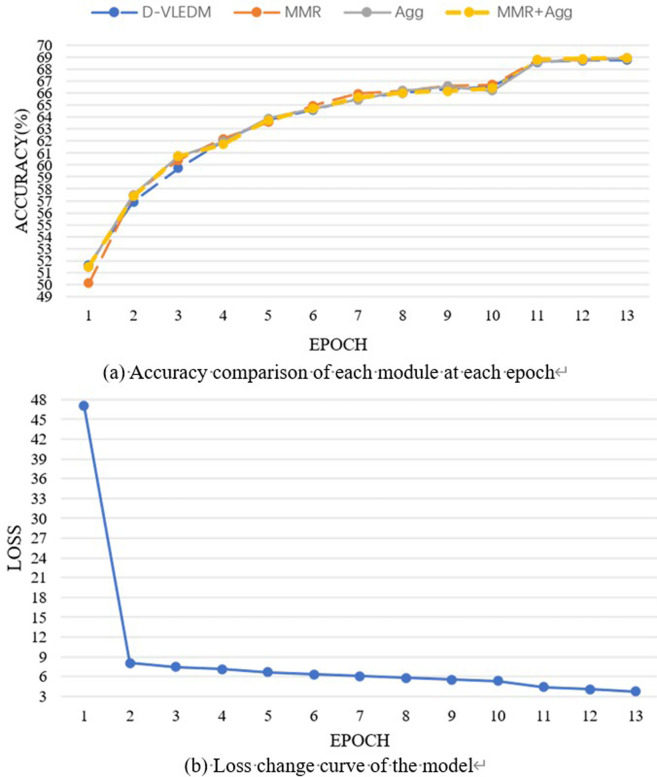
Accuracy and loss curves during training for each module.

In **Fig 14**, the combined MMR and Agg modules achieved optimal accuracy at epoch 13, with loss values stabilizing (larger loss values stem from sum aggregation across two GPUs). These results affirm the proposed method’s efficacy.

## 5. Conclusion

This study introduces a multimodal fusion VQA network based on Transformer architecture, designed to improve the representation of local visual features and comprehension of semantically integrated features. The model combines global-local attention, multimodal representation, and feature aggregation modules and employs three foundational attention units to construct a deep encoder-decoder, enhancing visual-language fusion for enriched semantic understanding. The multimodal representation module enables the network to focus on key query words, and image-guided question attention mitigates linguistic noise in the VQA task. Experimental results demonstrate the proposed model’s effectiveness in capturing fine-grained visual and language features, offering a novel approach to VQA model performance enhancement.

Future work, given the continuing advancement of AI and CV, will address (1) more effective multimodal fusion methods, (2) improved model interpretability, and (3) further optimizations for existing VQA algorithms.
